# Comprehensive ceRNA network for MACF1 regulates osteoblast proliferation

**DOI:** 10.1186/s12864-022-08910-0

**Published:** 2022-10-07

**Authors:** Shanfeng Jiang, Chong Yin, Kai Dang, Wenjuan Zhang, Ying Huai, Airong Qian

**Affiliations:** 1 Lab for Bone Metabolism, Xi’an Key Laboratory of Special Medicine and Health Engineering, Key Lab for Space Biosciences and Biotechnology, School of Life Sciences, Northwestern Polytechnical University, 710072 Xi’an, Shaanxi China; 2 Research Center for Special Medicine and Health Systems Engineering, School of Life Sciences, Northwestern Polytechnical University, 710072 Xi’an, Shaanxi China; 3 NPU-UAB Joint Laboratory for Bone Metabolism, School of Life Sciences, Northwestern Polytechnical University, 710072 Xi’an, Shaanxi China; 4 Department of Clinical Laboratory, Academician (expert) workstation, Lab of epigenetics and RNA therapy, Affiliated Hospital of North Sichuan Medical College, 637000 Nanchong, China

**Keywords:** Microtubule actin crosslinking factor 1 (macf1), Competing endogenous RNA (ceRNA), Osteoblast proliferation, LnRNA, MiRNA, Transcriptomic analysis

## Abstract

**Background:**

Previous studies have shown that microtubule actin crosslinking factor 1 (MACF1) can regulate osteoblast proliferation and differentiation through non-coding RNA (ncRNA) in bone-forming osteoblasts. However, the role of MACF1 in targeting the competing endogenous RNA (ceRNA) network to regulate osteoblast differentiation remains poorly understood. Here, we profiled messenger RNA (mRNA), microRNA (miRNA), and long ncRNA (lncRNA) expression in MACF1 knockdown MC3TC‑E1 pre‑osteoblast cells.

**Results:**

In total, 547 lncRNAs, 107 miRNAs, and 376 mRNAs were differentially expressed. Significantly altered lncRNAs, miRNAs, and mRNAs were primarily found on chromosome 2. A lncRNA-miRNA-mRNA network was constructed using a bioinformatics computational approach. The network indicated that mir-7063 and mir-7646 were the most potent ncRNA regulators and *mef2c* was the most potent target gene. Pathway enrichment analysis showed that the fluid shear stress and atherosclerosis, p53 signaling, and focal adhesion pathways were highly enriched and contributed to osteoblast proliferation. Importantly, the fluid shear stress and atherosclerosis pathway was co-regulated by lncRNAs and miRNAs. In this pathway, *Dusp1* was regulated by AK079370, while *Arhgef2* was regulated by mir-5101. Furthermore, *Map3k5* was regulated by AK154638 and mir-466q simultaneously. AK003142 and mir-3082-5p as well as Ak141402 and mir-446 m-3p were identified as interacting pairs that regulate target genes.

**Conclusion:**

This study revealed the global expression profile of ceRNAs involved in the differentiation of MC3TC‑E1 osteoblasts induced by MACF1 deletion. These results indicate that loss of MACF1 activates a comprehensive ceRNA network to regulate osteoblast proliferation.

**Supplementary information:**

The online version contains supplementary material available at 10.1186/s12864-022-08910-0.

## Background

Decreased bone formation plays a major role in osteoporosis, which results in low bone mass and increased fracture risk [1]. New bone formation is primarily mediated by osteoblasts[2, 3]. Therefore, regulating osteoblast proliferation and differentiation can enhance bone formation.

Previous studies have shown that microtubule actin crosslinking factor 1 (MACF1) can regulate osteoblast proliferation and differentiation in bone-forming osteoblasts [4, 5]. MACF1 can bind to actin filaments through its N-terminal calponin homology domain and positively regulates the Wnt/b-catenin signaling pathway, which is involved in multiple stages of osteoblast differentiation and bone formation [6–8]. To date, however, these studies have focused on a single regulator and systematic transcriptome-wide analysis of the MACF1 regulation network, especially the competing endogenous RNA (ceRNA) network, remains limited.

Non-coding RNA (ncRNA) is thought to play an important role in cellular processes. More recently, it has also been suggested that microRNA (miRNA) and long ncRNA (lncRNA) interact with each other, imposing an additional level of post-transcriptional regulation [9–11]. Furthermore, ncRNA and messenger RNA (mRNA) can form a well-regulated ceRNA interaction network. MiRNAs are a relatively well-documented class of ncRNAs involved in the regulation of various biological processes [12–15]. They can cause transcriptional degradation or translational inhibition by post-transcriptional regulation and binding to mature mRNAs. Many miRNAs have been implicated in the regulation of osteogenic differentiation. Mir-27a-3p and mir-365 can enhance osteogenesis in MC3T3-E1 cells, while mir-195, mir-146a, and mir-705 can inhibit this process [16–20]. LncRNAs, the largest class of ncRNAs in the mammalian genome, undergo further alteration by post-transcriptional modification to regulate gene expression [21]. Several studies have indicated that lncRNAs can promote osteoblast proliferation and differentiation by direct regulation of genes. For example, lnc-ob1 regulates osteoblast activity and bone formation in mice by up-regulating the osteogenic transcription factor Osterix [22]. In addition, H19 can act as a competitive inhibitor of mir-141 and mir-22 to reverse their inhibition of Wnt/beta-catenin signaling, thereby promoting osteogenic differentiation [23, 24].

To date, however, few studies have explored the pre-transcriptional levels of ncRNAs involved in osteogenesis. Furthermore, none of these interaction networks have been shown to regulate the MACF1-induced osteoblast proliferation of MC3T3-E1 cells. Analysis of the genetic factors that affect osteoblast proliferation and differentiation will provide valuable insight into bone diseases. In this study, MC3T3-E1 cells, which are precursor cells of osteoblasts, were used to examine the regulation pathways of MACF1.We conducted integrative analysis of the gene expression profiles of mRNAs, miRNAs, and lncRNAs induced by MACF1.

## Results

### Profiles of DEGs and ncRNAs

**Fig. 1:**
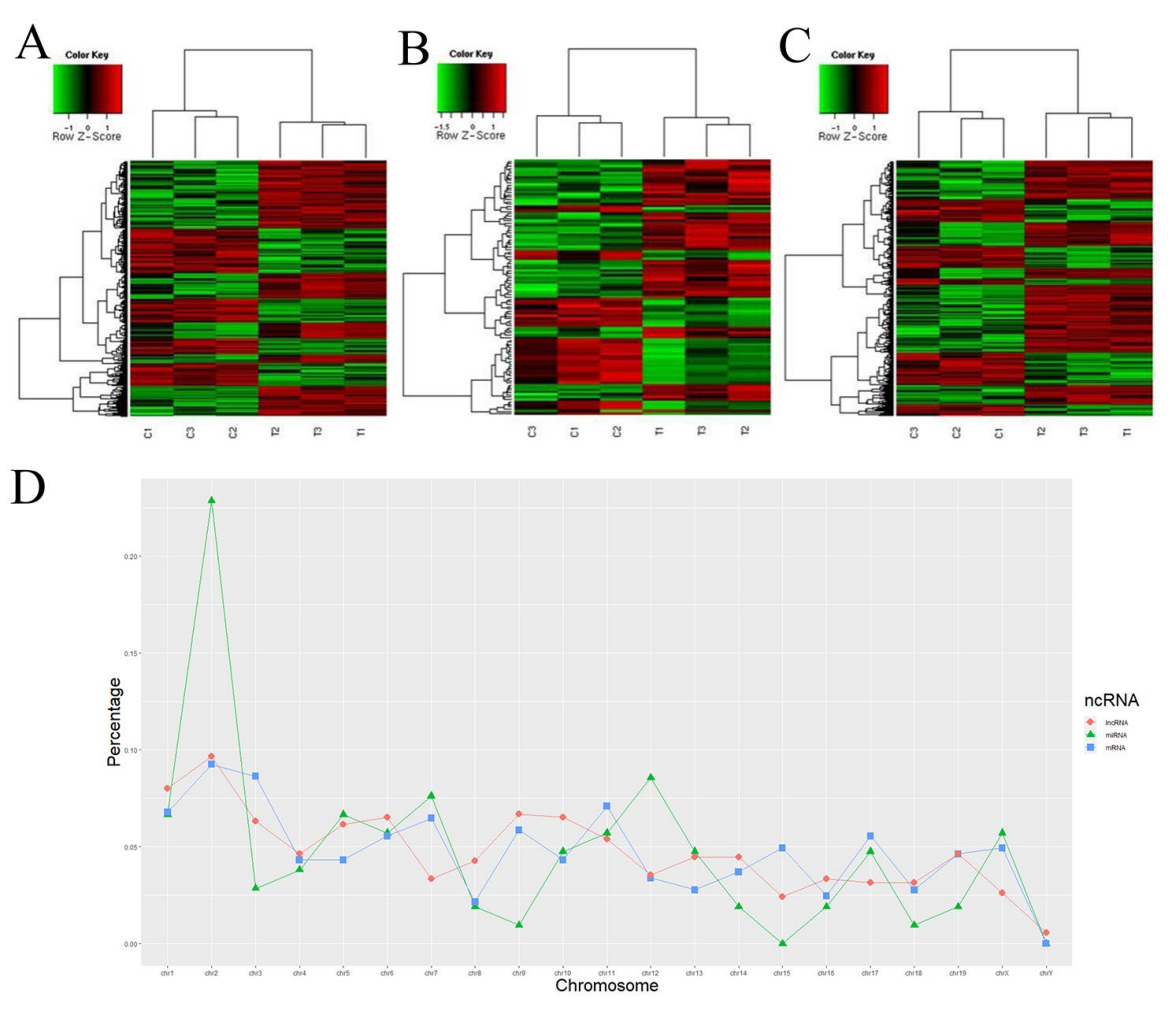
The profile of differentially expressed lncRNA, miRNA and mRNA in the MACF1 knockdown. MC3TC‑E1 cells. A: Hierarchical cluster analysis of the differentially expressed lncRNAs. The red color represented higher expression, and the green color represented lower expression. B: Hierarchical cluster analysis of the differentially expressed miRNAs. The red color represented higher expression, and the green color represented lower expression. C: Hierarchical cluster analysis of the differentially expressed mRNAs. The red color represented higher expression, and the green color represented lower expression. D: Location distributions of deregulated lncRNAs, miRNA and mRNAs on chromosomes

After applying a stringent filtering approach, we identified 547 differentially expressed lncRNAs in the MACF1-knockdown MC3T3-E1 cells, including 205 up-regulated and 304 down-regulated lncRNAs. We also identified 107 differentially expressed miRNAs, including 64 up-regulated and 43 down-regulated miRNAs, and 376 differentially expressed mRNAs, including 236 up-regulated and 140 down-regulated mRNAs, as presented in the clustering heat map (Fig. 1). Although the number of dysregulated miRNAs was the lowest, miRNAs had a higher ratio of DEGs/identified genes (5.63%) compared to lncRNAs (1.35%) and mRNAs (1.29%) (Table 1). The top three differentially expressed miRNAs were up-regulated mir-6942-3p and down-regulated mir-1950 and mir-669p-3p. AK033832 was the most up-regulated lncRNA and was located on chromosome 1. Locational distributions of the differentially expressed lncRNAs, miRNAs, and mRNAs were analyzed synchronously. The significantly altered lncRNAs, miRNAs, and mRNAs were found at the highest levels on chromosome 2 and at the lowest levels on chromosome Y (Fig. 1). No relatively consistent variation pattern was observed among the aberrant lncRNA and miRNA species. In addition, 25 lncRNAs, four miRNAs, and 14 mRNAs were located on chromosome 4, which was also the location of MACF1.

**Table 1:** Profiles of DEGs and ncRNAs in MACF1-knockdown cell line

Gene	Total number	Expression	MACF1^−/−^ vs. WT	Ratio
lncRNA	47 047	Up-regulated	369	0.78%
		Down-regulated	268	0.57%
miRNA	1 900	Up-regulated	64	3.37%
		Down-regulated	43	2.26%
mRNA	29 086	Up-regulated	236	0.81%
		Down-regulated	140	0.48%

MACF1^−/−^ : MACF1-knockdown cell line, WTL: wide type

## Comprehensive functional analysis of genes and ncRNAs

**Fig. 2:**
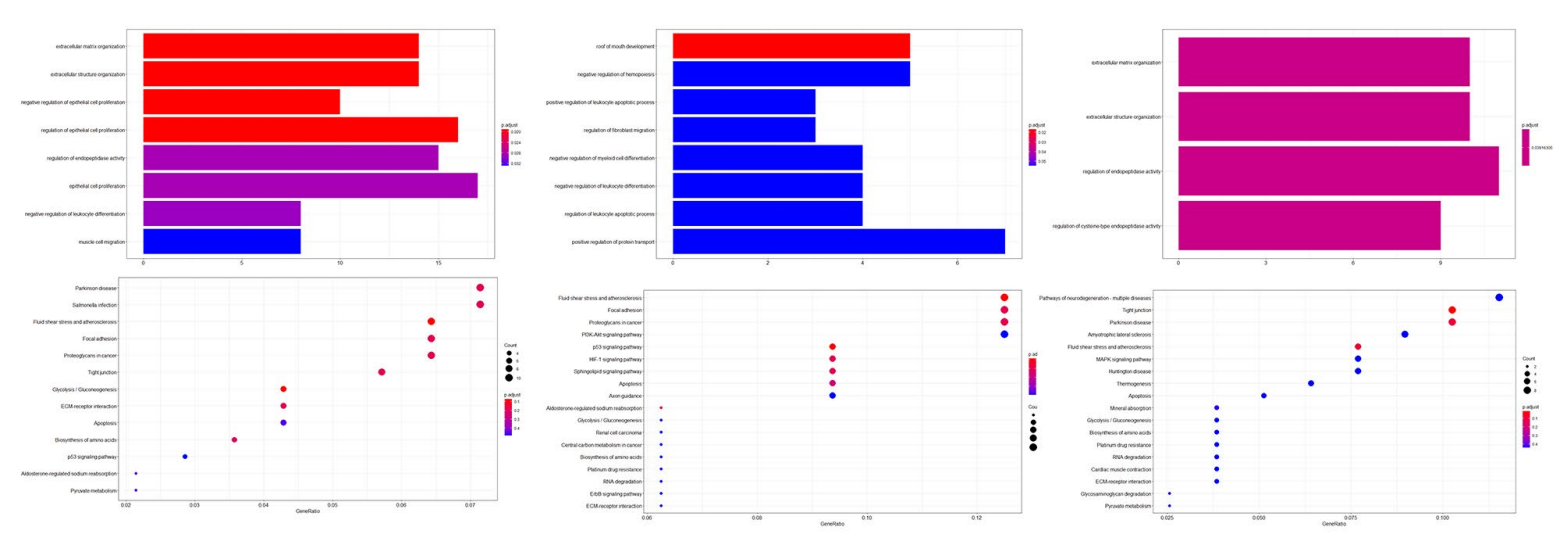
Functional enrichment analysis of ceRNAs. A: GO and KEGG analysis of differently expressed mRNA. B: GO and KEGG analysis of differently expressed genes targeted by miRNA. C: GO and KEGG analysis of differently expressed genes targeted by lncRNA.

The main functions of the differentially expressed mRNAs were explored using GO annotation and KEGG pathway enrichment analysis (Fig. 2). All enriched biological processes were related to organization, proliferation, migration, and differentiation (Fig. 2). For example, both regulation and negative regulation of epithelial cell proliferation were enriched, and extracellular matrix organization was the most enriched process. For pathway analysis, the fluid shear stress and atherosclerosis, p53 signaling, and focal adhesion pathways, which are involved in osteoblast proliferation [25–29], were all enriched.

For miRNA regulation analysis, biological processes related to migration and differentiation, which are influenced by MACF1 [30, 31], were significantly enriched. Among the miRNAs enriched in KEGG pathways, mir-370‐3p was predicted to have the greatest number of targets. Pathway analysis indicated no significant pathway was enriched in the miRNA-regulated genes.

We identified four candidate pathways related to osteoblast proliferation, i.e., fluid shear stress and atherosclerosis, p53 signaling, focal adhesion and PI3k-Akt pathways. A total of 22 miRNAs were involved in these pathways through regulating target genes. For example, six miRNAs (mir-7646-3p, mir-7063-3p, mir-669c-3p, mir-5101, mir-466 m-3p, and mir-466f-3p) were predicted to target *Mef2c* in the PI3K-Akt signaling pathway.

Only four biological processes were significantly enriched in the lncRNA-regulated mRNAs. Among those processes, extracellular matrix organization and extracellular structure organization are involved in the accumulation of cytoskeletal components [32]. Regarding enriched pathways in the lncRNAs, MAPK signaling and fluid shear stress were identified as two candidate pathways involved in the regulation of osteoblast proliferation.

**Fig. 3:**
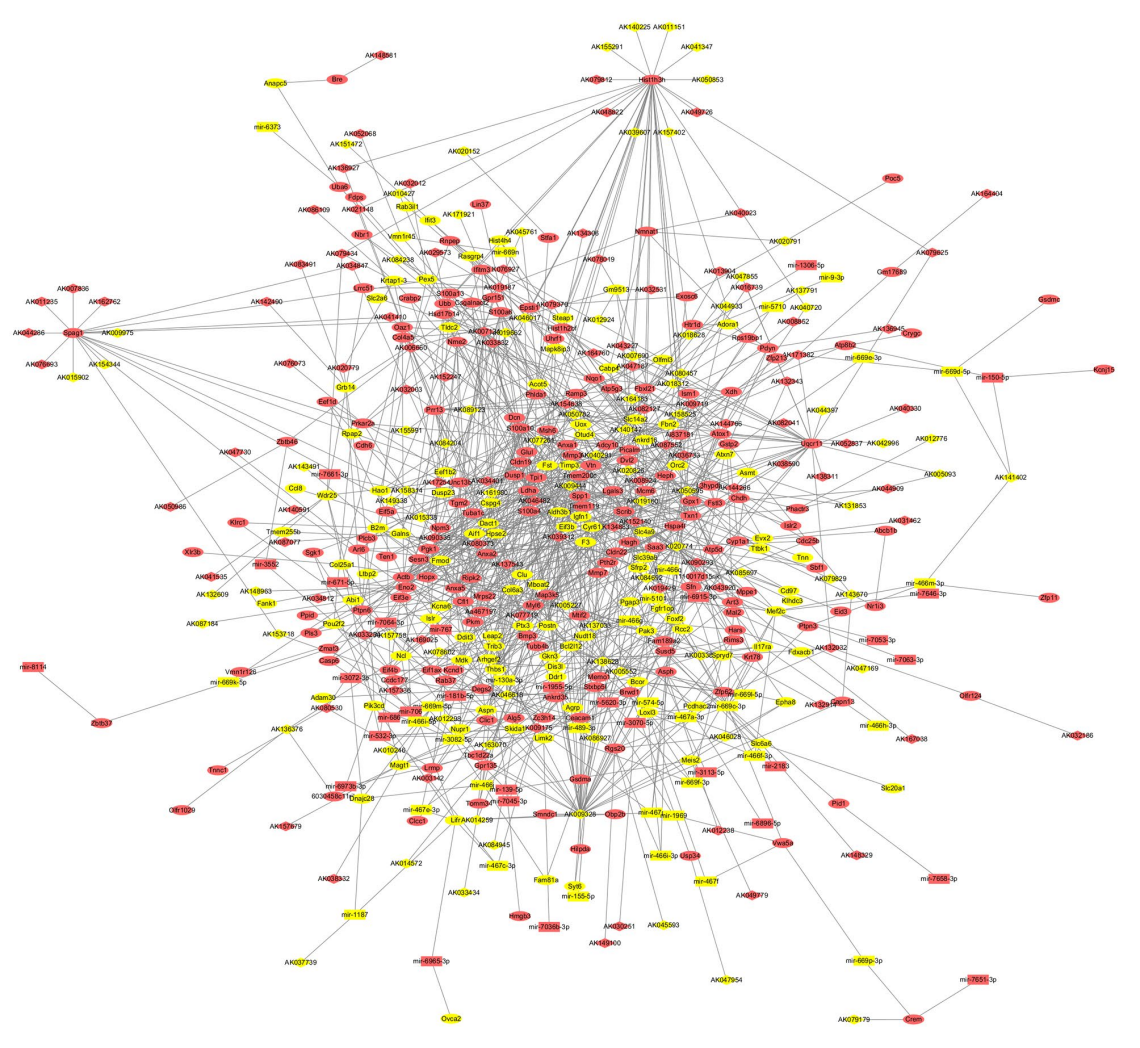
The connected interaction network of differently expressed ceRNA. The ellipse nodes represent miRNAs, the rectangle nodes represents mRNAs, and the diamond nodes represents lncRNAs. Red color indicated genes were up regulated and yellow indicated genes were down regulated

Of the 1030 dysregulated ceRNAs, 538 interacted with each other (Fig. 3). In addition, some isolated sub-networks only included two components. The core network consisted of 530 nodes and 1 196 connections among ceRNAs, including 181 lncRNAs, 63 miRNAs, and 286 mRNAs. Within this core network, 593 pairs showed positive regulatory associations and 603 pairs showed negative regulatory associations.

Results showed that the *Actb* coding gene had the highest degree among all mRNAs, while AK009328 had the highest degree among all dysregulated RNAs (Table 2). *Actb* also showed the highest closeness centrality and second highest betweenness centrality, indicating a core position in network topology. *Actb* is known to interact with certain genes related to cell proliferation, such as *Txn1*, *Casp6*, and *Arhgef2* [33–35]. Most miRNAs interacted with only 1–2 genes. Mir-669c-3p targeted 13 genes including *Mef2c*. AK009328 had 40 targets that are all coding genes.

**Table 2:** The top 10 hub genes with a high degree of connectivity

mRNA	degree	miRNA	degree	lncRNA	degree
Actb	23	mir-669c-3p	13	AK009328	40
Hist1h3h	20	mir-3082-5p	7	AK138628	27
Dcn	17	mir-181b-5p	5	AK020774	26
Lgals3	17	mir-466i-5p	5	AK050595	23
Anxa5	16	mir-467a-3p	5	AK090335	21
Hist1h3h	16	mir-532-3p	5	AK152140	19
Spp1	16	mir-669f-3p	5	AK007124	16
Tpi1	16	mir-130a-3p	4	AK046618	16
Thbs1	15	mir-466f-3p	4	AK087552	16
Tmem200c	14	mir-466j	4	AK019187	15

Degree is the measure of the total number of edges connected to a particular node

## Primary co-regulated pathway in ceRNA network

**Fig. 4:**
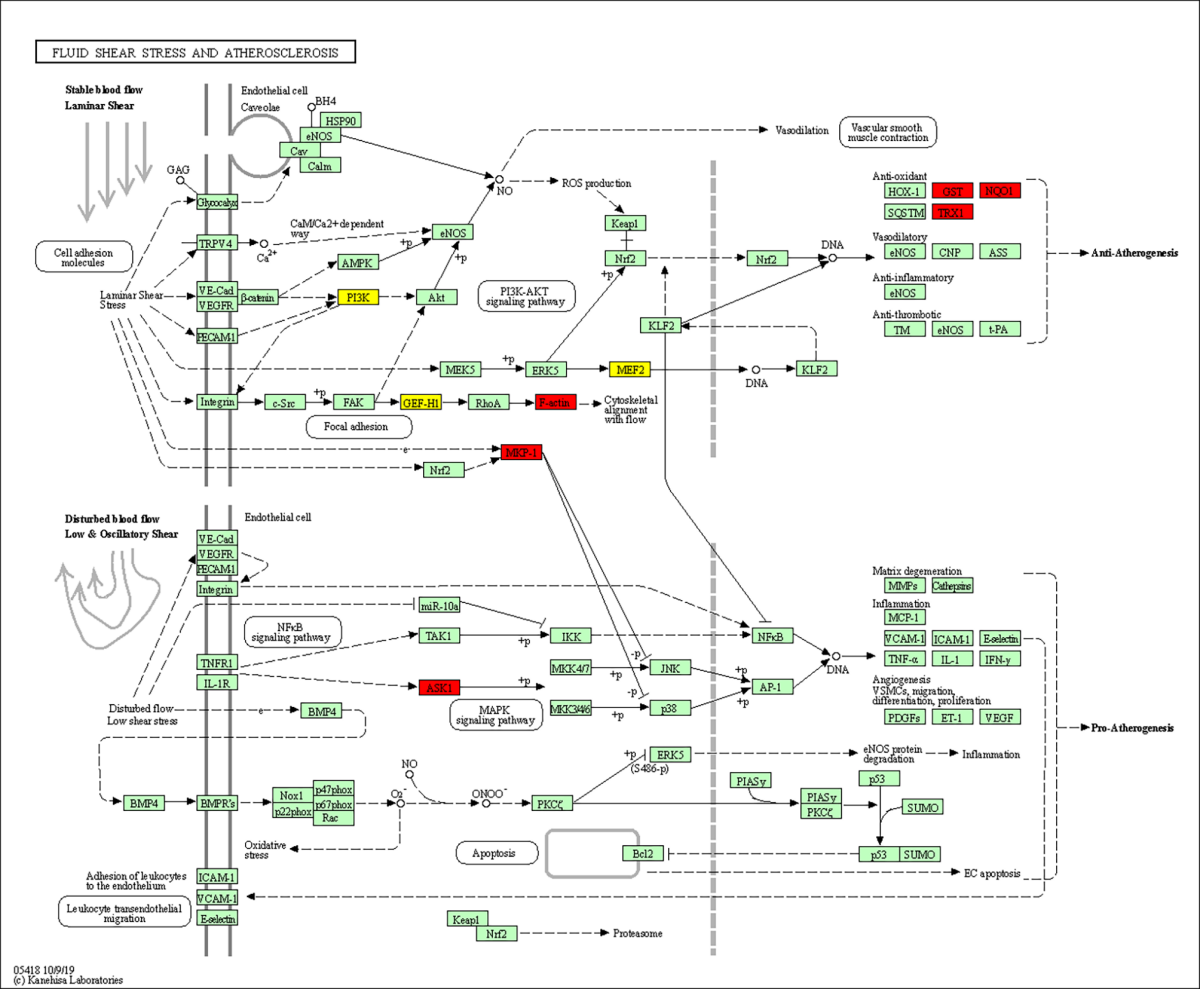
Fluid shear stress and atherosclerosis pathway. Red nodes indicated mRNA were up regulated and yellow nodes indicated mRNA were down regulated

After functional analysis, the fluid shear stress and atherosclerosis pathway was the most significantly enriched in the entire ceRNA network. Further expression analysis of this pathway was carried out, which identified six up-regulated genes and four down-regulated genes (Fig. 4). Notably, this pathway shares many processes with the focal adhesion, cell apoptosis, PI3k-Akt, MAPK, and NF-κB signaling pathways, which are all related to cell growth [36, 37]. For example, FAK, a key gene in the focal adhesion pathway, is a promoter of osteoblast proliferation [28, 29]. Our results also showed that *Mef2* and *PI3K* in the PI3k-Akt pathway were down-regulated, which may reduce cell proliferation [30]. The MAPK signaling pathway is activated in osteoblasts under fluoride exposure and can stimulate growth [31–33]. No dysregulated genes in the NF-κB signaling and apoptosis pathways were enriched.

## Co-modules of ceRNA related to the fluid shear stress and atherosclerosis pathway

After network-regularized sparse orthogonal-regularized joint non-negative matrix factorization (NSOJNMF), we obtained 200 ceRNA modules, each containing an average of 6.8 mRNAs, 1.7 miRNAs, and 7.9 lncRNAs. If more than one gene in a module participated in the fluid shear stress and atherosclerosis pathway, the module was considered to be associated with that pathway. For example, genes in co-module 15 involved in the fluid shear stress and atherosclerosis pathway included *Actb* and *Txn1*. Genes, lncRNAs, and miRNAs in the module were highly correlated (Fig. 4). Although other genes in the module are not involved in the fluid shear stress and atherosclerosis pathway, *S100a6* and *Ifitm3* are known to regulate PI3K signaling [38, 39], a sub-pathway of the fluid shear stress and atherosclerosis pathway. In addition, mir-466 h is implicated in apoptosis regulation [40].

## Predicted MACF1 regulation network for MC3T3‑E1 osteoblast proliferation

**Fig. 5:**
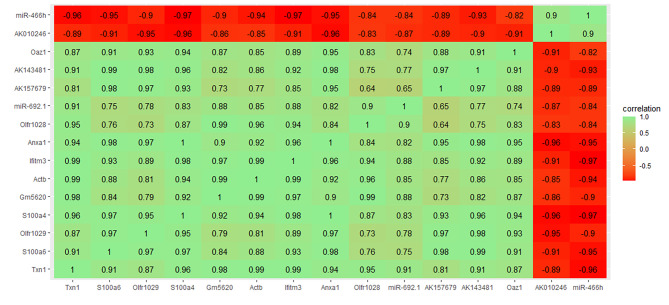
Spearman correlation of lncRNAs and mRNAs in ceRNA network. Green (red) indicates positive (negative) correlation

After analysis, we reconstructed the ceRNA network with genes and their interaction ncRNA pairs in the fluid shear stress and atherosclerosis pathway (Fig. 5). We found that MACF1 regulated miRNAs and proliferation-related genes via its targeted genes directly and indirectly. For example, knockdown of MACF1 attenuated the phosphorylation of GSK3β, which regulated transcription factors targeting miRNAs and, in turn, dysregulated the expression of core genes.

Our results also indicated that Mef2c plays an important role in the MACF1 regulation network. Firstly, mir-466 m-3p, mir-466f-3p, and mir-5101, which are predicated to inhibit *Mef2c*, were all down-regulated, as was AK141402, which can sponge mir-466 m-3p. In addition, mir-7646-3p and mir-7063-3p were up-regulated, consistent with the down-regulation of *Mef2c*. In mammalian cells, p38 can be regulated by dysregulated MAP3Ks and DUSP1 [41, 42], and p38-catalyzed phosphorylation can increase the transactivation of MEF2C [43, 44].

**Fig. 6:**
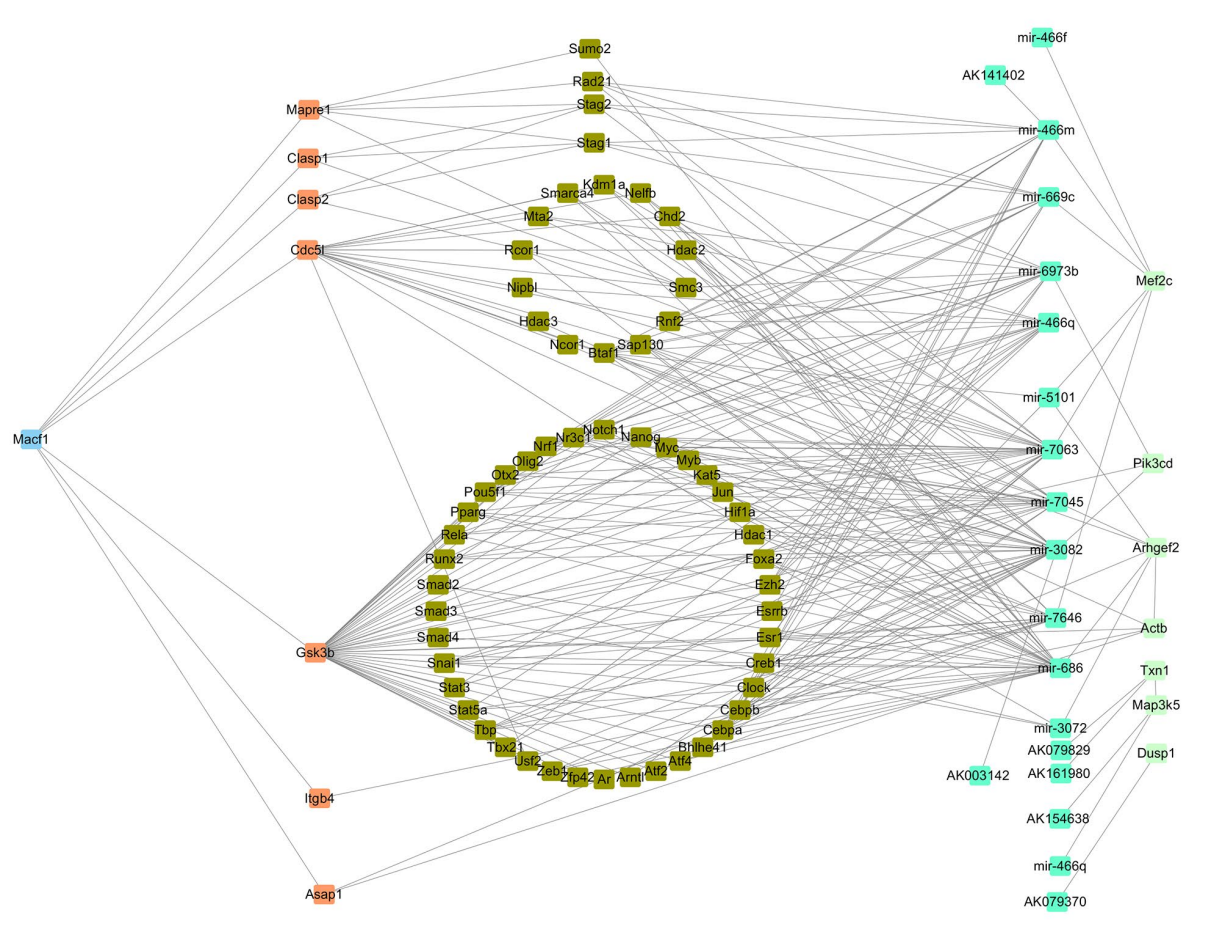
Predicted MACF1 regulation network of ceRNA in MC3T3-E1 cells. Orange nodes indicate genes interact with MACF1 directly; olive nodes indicate transcription factors that regulated related miRNAs; aquamarine nodes indicate ncRNA and lightgreen nodes indicate genes enriched in fluid shear stress and atherosclerosis pathway

## Discussion

MACF1 plays an important role in regulating osteoblast proliferation and differentiation in bone-forming osteoblasts [4, 5, 45]. However, no integrated network has been reported regarding its underlying regulatory mechanism.

In recent years, ncRNAs, such as lncRNAs and miRNAs, have emerged as previously underappreciated classes of gene expression modulators that regulate various cellular processes [9–11, 46]. In the current study, we illustrated a comprehensive ceRNA network for MACF1 deletion to regulate osteoblast proliferation through bioinformatics analysis of gene chip data.

In the current study, MACF1 deletion resulted in a large number of differentially expressed RNAs. The most enriched GO terms in the mRNAs and lncRNAs were related to positive regulation of cell death and extracellular matrix organization, which may be related to MACF1 deletion as it binds with F-actin and microtubules [7]. Based on mRNA analysis, the fluid shear stress and atherosclerosis, p53 signaling, and focal adhesion pathways promoted osteoblastogenesis. Fluid shear stress plays a critical role in promoting osteoblast proliferation and differentiation [47, 48]. It can promote cytoskeletal reorganization to activate the ERK5 pathway [25, 26, 47]. MACF1 deletion may result in similar cytoskeletal reorganization to regulate osteoblast proliferation. Among the 19 genes mapped to the three pathways in the current work, *Map3k* and *Mef2* are well-studied in osteoblast proliferation [49–52]. *Map3k* participates in MEK1 and MEK2 activation [53]. In turn, MEK1/2 activate ERKs to phosphorylate RUNX2, thereby enhancing the proliferation of osteoblast progenitors. *Mef2* is a component of the enhanceosome that regulates the enhancer of *Runx2* [54]. In addition, the *Txn1*, *Dusp1*, *Nqo1*, *Arhgef2*, *Pik3cd*, *Sfn*, and *Sesn3* genes can regulate cell proliferation too.

Five pathways promoting osteoblastogenesis were found to be regulated by miRNAs. In addition to the fluid shear stress and atherosclerosis pathway, the p53 signaling and focal adhesion pathways were also enriched in differentially expressed mRNAs. Furthermore, the apoptosis and PI3K-Akt signaling pathways were also enriched in the miRNA target genes. Several miRNAs targeting these pathways are reported to influence cell proliferation. For example, although Mir-532-3p inhibits osteogenic differentiation in MC3T3-E1 cells [55], it also inhibits proliferation by regulating β-catenin expression and targeting the phosphatase and tensin homolog (PTEN) gene in the PI3K/AKT signaling pathway [56]. Thus, Mir-532-3p may be a potential regulating factor of osteoblast proliferation following MACF1 deletion. In addition, Mir-466f-3p is down-regulated during osteoblast differentiation and bone regeneration [57] and Mir-139-5p can inhibit mesenchymal stem cell (MSC) osteogenesis through the Wnt/β-catenin pathway by directly targeting CTNNB1 and frizzled 4 (FZD4) [58]. Thus, these miRNAs may also participate in cell proliferation induced by MACF1. We also identified several dysregulated miRNAs that are involved in cell proliferation, although they have not been reported in osteogenesis. For example, Mir-466q modulates the p38 MAPK signaling pathway by inhibiting the expression of its target gene *Map3k* [59]. Mir-574-5p targets *Bcl11a* and *Sox2* to attenuate proliferation in triple-negative breast cancer cells and governs cell proliferation through the Wnt/β-catenin pathway in PTC-1 cells [60]. Mir-489-3p can also inhibit cell proliferation by targeting the brain-derived neurotrophic factor-mediated PI3K/AKT pathway in glioblastoma cells and suppress proliferation by targeting JAG1 in bladder cancer cells [61]. For some miRNAs, such as mir-7646-3p, mir-7045-3p, and mir-6973b-3p, although they have not been implicated in regulating proliferation, our results indicate that they have the potential to influence osteogenesis through their target genes.

The fluid shear stress and atherosclerosis, MAPK signaling, and apoptosis pathways can influence proliferation via lncRNA regulation [26, 47, 48, 53, 62]. Here, four lncRNAs (AK079829, AK079370, AK154638, and AK161980) were predicted to regulate these pathways. Previous research has shown that AK079370 can inhibit bone formation by suppressing the Wnt/β-catenin pathway [63]. Our results also indicated that AK079370 may interact with *Dusp1*, which can inhibit cell proliferation via the ERK signaling pathway [64]. Two cell proliferation genes,T*xn1* and *Map3k5*, were also regulated by AK079829 and AK161980 and by AK154638, respectively. However, their functional mechanisms related to osteoblasts require further experimental validation.

We also identified three interaction pairs (Mir-3082-5p and AK003142, mir-466 m-3p and AK141402, and mir-532-3p and AK009175) that may regulate osteoblastogenesis-related genes. As mentioned above, mir-532-3p has the capacity to inhibit proliferation by regulating β-catenin expression, while mir-466 m-3p is predicted to target *Mef2c* in the PI3K-Akt signaling pathway [56]. In our study, Mir-3082-5p was predicated to target *Pik3* to participate in osteogenesis. However, although several lncRNA-mRNA pairs were identified, none directly participated in proliferation. Thus, lncRNAs appear to regulate osteoblastogenesis by sponging miRNAs rather than by directly regulating mRNAs.

Co-module analysis also revealed that ceRNA network contributed to the cell proliferation. A total of 106 co-modules were found related to fluid shear stress and atherosclerosis pathway. This may be caused by the high degree of connectivity of *Actb*, which is involved in the co-modules and the fluid shear stress and atherosclerosis pathway simultaneously. *Actb* also participates in focal adhesion and adherent junction pathways, two key regulation pathways in osteoblast proliferation, Besides, *Txn1* is involved in co-modules 15,100 and 187. *Txn1* may play an important role in regulating cell proliferation [65]. More importantly, the analysis of co-modules is convenient to find RNAs that could complement the ceRNA regulation network. For example, miR-466 in co-module 187 could significantly inhibit cell proliferation while miR-671-5p in co-module 100 could foster the proliferation [66–68]. The results indicate that ceRNA network plays an important role in the regulation of cell proliferation.

We found that the fluid shear stress and atherosclerosis pathway was enriched in mRNAs, lncRNAs, and miRNAs simultaneously. Fluid shear stress is thought to mediate bone cell proliferation by producing cellular chemical signals [47]. The extracellular signal-regulated kinase 5 (ERK5) pathway is well-studied in regard to the promotion effects of fluid shear stress on osteoblast proliferation [25, 26, 69]. The fluid shear stress and atherosclerosis pathway shares various processes with the PI3k-AKT signaling, focal adhesion, NF-κB signaling, MAPK signaling pathways, which are key regulation pathways in osteoblast proliferation [27, 28, 49, 50, 52, 53, 70–72]. After we reconstructed the lncRNA and miRNA-regulated pathway for DEGs, all related lncRNAs increased. Core genes in this interaction network included *Pik3ca*, *Mef2a*, *Map3k5*, and *Dusp1*, which are related to cell proliferation [64, 73, 74]. The DEGs revealed that the network contributed to osteoblast proliferation via multiple approaches. For example, AK154638 and AK079370 were predicted to act as antisense lncRNAs for *Map3k5* and *Dusp1*, respectively, to inhibit osteoblast proliferation. Mir-466f-3p, mir-510, mir-466 m-3p, mir-669c, mir-7063, and mir-7646 negatively regulated osteogenesis by binding to *Mef2c*, while AK141402 sponged mir-466 m-3p to resist this inhibition. In previous research, we found that proliferation and differentiation are inhibited in MACF1-knockdown MC3T3-E1 cells [4, 30]. This is consistent with the function of *Mef2c* in cell proliferation and differentiation, which regulates a novel *Runx2* enhancer for osteoblast-specific expression [54]. In our regulation network, only mir-7063 and mir-7646 targeting *mef2c* were increased. Thus, it is possible that MACF1 influences *Mef2c* expression via GSK3β and cell division cycle 5 like (CDC5L), which may target miRNA transcription factors.

Overall, our analysis revealed a comprehensive ceRNA network of MACF1 for the regulation of osteoblast proliferation. Using bioinformatics analysis, a considerable number of functional ncRNAs were predicted to be involved in the regulation of osteoblast proliferation. The fluid shear stress and atherosclerosis pathway was presumed as the most important pathway for MACF1 to regulate osteogenesis. Although further in vivo and in vitro experiments are required to test this hypothesis, the present study provides novel insight into the molecular mechanism underlying osteoblast proliferation.

## Materials and methods

### Cell culture and construction of MACF1-knockdown cell line

Murine preosteoblast MC3T3-E1 cell line was generously provided by Dr. Hong Zhou (University of Sydney, Sydney, NSW, Australia). The MC3T3-E1 cells were cultured in α-modified Eagle’s medium (α-MEM) (Gibco, Carlsbad, CA, USA) supplemented with 10% fetal bovine serum (FBS) (Life Technologies, USA) and 1% penicillin/streptomycin. Cells were incubated for 15 days at 37 ℃ with 5% CO_2_ in a humidified chamber. The MACF1-knockdown cell line was constructed as described in our previous report [4]. In brief, the MC3T3-E1 cells were transfected with short hairpin RNA (shRNA) specifically targeting the murine MACF1 lentivirus vector or with scrambled shRNA, and the stably transfected cell lines were selected using puromycin. After 15 days of selection, all cells were collected for further study.

## LncRNA, miRNA, and mRNA microarray analyses

The collected MC3T3-E1 cells were subjected to sequencing by RiboBio Co., Ltd. (Guangzhou, China) for lncRNA, miRNA, and mRNA microarray analyses. Total RNA was harvested and quantified, and its quantity and purity were assessed using a K5500 Micro-Spectrophotometer (Beijing Kaiao Technology Development Co., Ltd., Beijing, China). Here, A260/A280 ≥ 1.5 and A260/A230 ≥ 1 indicated acceptable RNA purity and RNA integrity number (RIN) ≥ 7 (based on an Agilent 2200 RNA assay, Agilent Technologies, USA) indicated acceptable RNA integrity. Genomic DNA contamination was evaluated by gel electrophoresis.

## Differentially expressed mRNAs, miRNAs, and lncRNAs

The fold-change of each differentially expressed mRNA and lncRNA was obtained by log2 fold-change (normalized spot intensities were transformed to gene expression log2 ratios between test and control samples). The *P*-values were calculated using analysis of variance (ANOVA). Differentially expressed genes (DEGs) were determined based on fold-change > 2 and adjusted *P* < 0.05.

For lncRNA identification, the transcripts mapped to known genes were eliminated. The Coding Potential Calculator (CPC) and Coding Non-Coding Index (CNCI) were then used to predict the coding potential of the sequences, requiring CPC and CNCI scores < 0 as indicators for potential lncRNAs.

Finally, differentially expressed mRNAs were selected for cluster analysis performed using the R language package ggplots (v3.3.2) according to Fragments Per Kilobase of exon model per Million mapped fragments (RPKM) values.

## Prediction of target genes of differentially expressed ncRNAs

Based on the co-expression of lncRNAs and mRNAs (correlation 0.99 or − 0.99 and *P* < 0.05), the functions of the lncRNAs were executed on coding genes via cis- or trans-regulation. The lncRNAs and target coding genes were considered lncRNA-mRNA pairs. BEDTools (v2.29.1) was used for positional relationship analysis. If the lncRNA gene was within 100 kb upstream or downstream of the coding gene, it was determined to be cis-regulatory, while trans-prediction was based on the binding energy of the lncRNA and coding genes according to sequence complementarity. Pairs of lncRNA and mRNA with a binding ndG < − 0.1 based on LncTar analysis were deemed interactive.

Target gene prediction for miRNAs was performed using the Encyclopedia of RNA Interactomes (ENCORI) database [75], which provides seven miRNA target gene prediction programs, i.e., PITA, RNA22, miRmap, DIANA-microT, miRanda, PicTar, and TargetScan. The prediction results were screened using at least three program predictions.

To identify the miRNAs that can target lncRNAs, the binding of lncRNAs to miRNAs was predicted using the bioinformatics tool starBase with ENCORI APIs [75].

## Construction and analysis of lncRNA, miRNA, and mRNA interaction network

LncRNAs can target mRNAs through cis or trans activity. Coupled with the targeted relationship between miRNAs and mRNAs and possible targeted relationship between miRNAs and lncRNAs, lncRNA-miRNA-mRNA network interactions were identified using STRING (v11). Results were visualized using Cytoscape (v3.6.0) [76]. In the network diagram, connections indicate possible regulatory relationships. Core genes were detected using NetworkAnalyzer (v2.8) by calculating network topology parameters.

## Functional enrichment analysis

To determine the functional modules, we focused on the DEGs and ncRNAs and conducted functional enrichment analysis with clusterProfiler [77]. For ncRNAs, target genes were used for analysis. Gene Ontology (GO) and Kyoto Encyclopedia of Genes and Genomes (KEGG) pathway enrichment analyses were screened at *P* < 0.05 and q-value < 0.05.

## Recognition of ceRNA co-modules

After identifying functional modules, to further discover their potential biological associations, we applied network-regularized sparse orthogonal-regularized joint non-negative matrix factorization (NSOJNMF) to identify correlative modules using multi-dimensional genomics data [78–80]. The code is public available at https://github.com/JN-WYJ/NSOJNMF. Briefly, the three microarray data X1, X2 and X3 were decomposed into a common basis matrix W ∈ R^m×K^, and different coefficient matrices Hi ∈ R^K×n^_i_ (i = 1,2,3) using the JNMF framework. The prior knowledge of the algorithm combination includes the known or predicted interactions of the three RNAs as described in the previous section. Z-score of each column was used in the coefficient matrix to select the members. Eventually, k ceRNA co-modules can be identified. K was assigned to 200 according to the number of the mRNA enrichment pathways in this study. The constraint parameters were λ1 = λ2 = 0.001. The sparse parameter γ = 10 and maximum number of iterations to run is 500. Matlab (R2021a) was used to calculate the co-modules, with the selection of co-module number set to 200. The constraint parameters were λ = 0.001 and γ = 10.

## Re-construction of ceRNA regulation pathway for proliferation in MACF1 deletion cells

To build a MACF1-regulated ceRNA network, STRING and TransmiR (v2.0) were used to predict the relationship between the core ceRNAs and MACF1. Firstly, direct target genes of MACF1 were extended by STRING and transcription factors of miRNA were predicted by TransmiR, respectively. The MACF1-target network and TF-miRNA network were then integrated into a sub-network of a previously built ceRNA network of core genes. Thus, a MACF1-TF-miRNA-mRNA network was constructed.

## Electronic supplementary material

Below is the link to the electronic supplementary material.

Supplementary Material 1

## Data Availability

mRNA and lncRNA arrays data have been deposited in the ArrayExpress database at EMBL-EBI (www.ebi.ac.uk/arrayexpress)under accession number E-MTAB-11,425 and E-MTAB-11,426, respectively. miRNA data is available at: https://www.ncbi.nlm.nih.gov/geo/query/acc.cgi?acc=GSE202962 (reviewer token: cvalwmqkbhsdbqp). The datasets used and/or analyzed during the current study are available from the corresponding author on reasonable request.
